# Effect of Behaviorally Designed Gamification With a Social Support Partner to Increase Mobility After Hospital Discharge: A Randomized Clinical Trial

**DOI:** 10.1001/jamanetworkopen.2021.0952

**Published:** 2021-03-24

**Authors:** S. Ryan Greysen, Sujatha Changolkar, Dylan S. Small, Catherine Reale, Charles A. L. Rareshide, Ashley Mercede, Christopher K. Snider, Heather M. Greysen, Rebecca Trotta, Scott D. Halpern, Mitesh S. Patel

**Affiliations:** 1 Section of Hospital Medicine, University of Pennsylvania Perelman School of Medicine, Philadelphia; 2 Center for Evidence-based Practice, University of Pennsylvania Perelman School of Medicine, Philadelphia; 3 University of Michigan School of Medicine, Ann Arbor; 4 The Wharton School, University of Pennsylvania, Philadelphia; 5 Crescenz Veterans Affairs Medical Center, Philadelphia, Pennsylvania; 6 University of Pennsylvania School of Nursing, Philadelphia; 7 Hospital of the University of Pennsylvania, Philadelphia; 8 Palliative and Advanced Illness Research Center, University of Pennsylvania, Philadelphia

## Abstract

**Importance:**

Hospitalization is associated with decreased mobility and functional decline. Behaviorally designed gamification can increase mobility in community settings but has not been tested among patients at risk for functional decline during a high-risk transition period after hospitalization.

**Objective:**

To test a behaviorally designed gamification intervention with a social support partner to increase patient mobility after hospital discharge.

**Design, Setting, and Participants:**

This study is a randomized clinical trial of a 12-week intervention without follow-up. Enrollment occurred from January 2018 to June 2019 at a referral hospital with a remote at-home monitoring intervention among patients living predominantly in 3 states (Pennsylvania, New Jersey, and Delaware). Participants included adult patients discharged from general medicine and oncology units to home. Data analysis was performed from October 2019 to March 2020.

**Interventions:**

All participants received a wearable device to track daily steps. The control group received feedback from the device but no other interventions. The intervention group entered into a 12-week game informed by behavioral economics to assign points and levels for achieving step goals and reinforced by a support partner who received updates on participant progress.

**Main Outcomes and Measures:**

The primary outcome was change in mean daily steps from baseline through the 12-week intervention. Secondary measures were change in functional status and urgent care utilization (ie, emergency department visits and hospital readmissions) within this period.

**Results:**

A total of 232 participants were enrolled in the study (118 randomized to control and 114 randomized to the intervention). Participants had a mean (SD) age of 40 (14) years, 141 (61%) were female, 101 (43%) were White, and 103 (44%) had an annual household income less than $50 000. Daily step counts increased from 3795 to 4652 steps (difference, 857 steps; 95% CI, 488 to 1224 steps) among intervention participants and increased from 3951 to 4499 steps (difference, 548 steps; 95% CI, 193 to 903 steps) among control participants. The change in mean daily step count from baseline was not significantly different for participants in the intervention group vs the control group (adjusted difference, 270 steps; 95% CI, −214 to 754 steps; *P* = .27). Among the subgroup of 76 participants with higher levels of social engagement, post hoc exploratory analyses showed a significant increase in mobility for intervention vs control (adjusted difference, 1125 steps; 95% CI, 409 to 1841 steps; *P* = .002). Fewer participants in this subgroup experienced functional decline (1 of 36 participants [4%] in the intervention group vs 5 of 40 participants [12%] in the control group) and hospital readmission at 30 days (3 of 36 participants [8%] in the intervention group vs 6 of 40 participants [15%] in the control group), but the differences were not statistically significant. There were no significant differences in these secondary outcomes for the overall sample.

**Conclusions and Relevance:**

Gamification with social incentives did not affect mobility or functional decline in all participants, but post hoc analysis suggests positive findings for both outcomes for patients with higher social engagement.

**Trial Registration:**

ClinicalTrials.gov Identifier: NCT03321279

## Key Points

**Question:**

Is behaviorally designed gamification with a social support partner associated with increased mobility and reduced functional decline among patients discharged from the hospital?

**Findings:**

In this randomized clinical trial of 232 adult patients hospitalized in general medicine and oncology units, gamification with social incentives did not increase postdischarge mobility (steps per day) or functional decline compared with control during the 12-week intervention. However, the subgroup of 76 patients with higher social engagement at baseline had an increase in mobility and a decrease in functional decline.

**Meaning:**

Gamification with social incentives did not affect mobility or functional decline in all participants but may be beneficial for patients with higher levels of social engagement.

## Teaser

This randomized clinical trial tests a behaviorally designed gamification intervention with a social support partner to increase patient mobility after hospital discharge.

## Introduction

Low mobility or limited walking is a key factor in functional decline, disability, and urgent care utilization^1,2^ and is often precipitated by hospitalization; approximately 1 in 3 adults with multiple chronic medical conditions have decreased mobility at the time of discharge.^3^ Mobility disability (defined as serious difficulty walking or climbing stairs) affects 31 million Americans^4^ and is associated with loss of independence,^5,6^ higher health care costs,^7,8^ and early mortality.^9,10^ Observational studies have demonstrated that patients who walk more during hospitalization have lower rates of functional decline,^11^ discharge to long-term facilities,^12^ and readmissions.^13^ Although low mobility during hospitalization is observed across most diagnoses and all ages, patients admitted for nonsurgical care^14^ who are older^15^ or middle-aged^16^ are at higher risk of these adverse outcomes.

Much less is known about walking during transitions of care and the immediate postdischarge period. Recent trials^17,18^ of multicomponent exercise programs after hospital discharge have demonstrated less functional decline and fewer rehospitalizations; however, adherence to nonwalking components was low, suggesting that walking may be the key factor. Moreover, the lack of adaptive goal setting, individualized feedback, and a behavioral change framework may also attenuate participant success. We have applied concepts from behavioral economics^19,20^ and gamification that address these gaps and have significantly increased walking among families in the Framingham Heart Cohort^21^ and among employees of a large consulting firm from 40 states.^22^ These methods, however, have not previously been applied to recently hospitalized patients at risk for low mobility and disability.

Recognizing the importance of social networks in these interventions,^23^ we designed a randomized clinical trial in a population of patients admitted to general medicine or oncology units that combined behaviorally designed gamification with a social support partner to promote walking after hospital discharge. Social support may be especially powerful for hospitalized patients because social networks are often activated in the setting of acute illness.^24,25^ We hypothesized that patients who received the intervention would show increased postdischarge mobility, decreased functional decline, and decreased urgent care utilization during the 12-week intervention period.

## Methods

### Study Design

The MOVE IT (Mobility and Outcomes for Validated Evidence–Incentive Trial) study was a randomized clinical trial conducted between January 1, 2018, and June 30, 2019, consisting of a 1-week run-in period and a 12-week intervention period. There was no follow-up period after the end of the intervention. Details of the study design and protocol have been published previously.^26^ The trial protocol (Supplement 1) was approved by the University of Pennsylvania institutional review board. This trial followed the Consolidated Standards of Reporting Trials (CONSORT) reporting guideline.

Each day, hospitalized patients were screened and approached in person. After providing informed consent (participants provided consent on a study tablet, where they had to check a box affirming they understood the study, giving consent, and then typing their name), eligible participants received a wrist-worn wearable device (Withings Activite Steel) that lasts about 6 months without charging. Our prior work^27^ has demonstrated these types of devices are accurate for tracking step counts and have been used successfully in recent interventions.^21,28,29^ We also ensured that participants were able to connect their device to the Way to Health platform,^30^ a research technology platform that automatically pulls data from the wearable device and sends prespecified text messages back to participants. We have used Way to Health successfully in several previous remote-monitoring and physical activity interventions.^21,22,28,31^

### Participants

**Figure 1.**
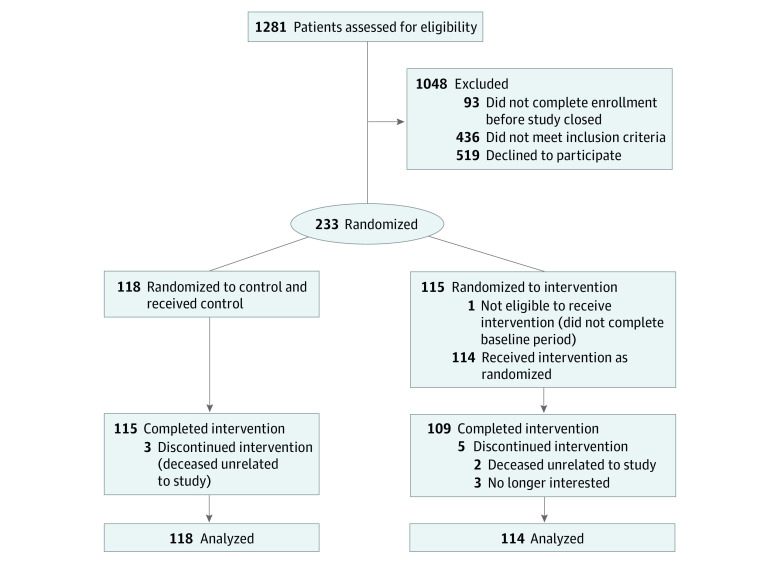
CONSORT Diagram Participants in all groups used a wearable device to track daily step counts, established a baseline, and selected a step goal increase. Participants in the control group received regular feedback from the wearable device and its smartphone application, but no other interventions. Participants in the intervention group received gamification with a social support partner that ran automatically for 12 weeks starting 1 week after hospital discharge.

Participants were recruited from 4 inpatient general medicine and 3 oncology units at the Hospital of the University of Pennsylvania. Participants were eligible if they were aged 18 years or older, able to speak English, provided informed consent, ambulated independently (Activity Measure for Post-Acute Care^32^ score ≥12), and owned a smartphone or tablet compatible with the wearable device. Participants were excluded according to the clinical judgment of the bedside nurse, current enrollment in another activity study, or any medical or other reason the participant would be unable to complete the 12-week trial (Figure 1). Participants also coenrolled in the RETAIN (Randomized Evaluation of Trial Acceptability by INcentive) study^33^ to evaluate the impact of financial incentives on participant enrollment and retention in a low-risk, randomized clinical trial. As part of the RETAIN study, all participants received $150 for enrolling in the trial and $150 for completing the 12-week intervention and follow-up surveys. Once enrolled, participants complete a series of surveys and validated questionnaires (eTable 1 in Supplement 2).

### Baseline Step Count and Randomization

The first week after discharge was a run-in period to establish baseline step count using 4 or more days of data capture and ignoring any days with fewer than 1000 steps because these values are unlikely to represent capture of actual activity during the whole day, according to previous work.^21,22,28,31^ After establishing a baseline, participants were randomized electronically to control or intervention with 1:1 allocation by stratifying on hospital unit and baseline steps (≤5000 steps per day, 5001-7500 steps per day, and >7500 steps per day) and using block sizes of 2. At the time of randomization, participants were notified by text and email with instructions for the 12-week study period that were specific to intervention and control but were not told which group they were in. All investigators, statisticians, and data analysts were blinded to group assignments until the study and analysis were completed.

### Step Goals

Steps were monitored passively in the control group by the wearable device, but participants were not informed of their baseline step count or any specific goal. All wearable devices had a factory preset goal of 10 000 steps per day, which could be adjusted by the participant. Each participant in the intervention group was informed of baseline step count and then given a new goal each week with 10% increase. This increase in goal is based on a recent successful trial by our group^28^ and was triggered only if the participant met their goal for the prior week by achieving 40 points for that week (discussed later).

### Interventions

Intervention participants entered into a mobility game with points and levels that was run automatically (participants need only strive for step goals) and received a daily notification on their progress. We leveraged social networks to increase participant accountability, sense of accomplishment, and overall engagement in this mobility game. Participants identified a family member or friend to be a support sponsor who was asked via email at the start of the study to provide support and encouragement to the participant to help them achieve their mobility goals. This partner received a weekly email update report on participant performance including points and level. The effectiveness of gamification and social support to change behavior can be enhanced by leveraging behavioral concepts.

Gamification in this study was designed using several core principles from behavioral economics.^21,22^ First, participants in the gamification group signed a precommitment pledge to strive to achieve their step goal during the study. Precommitment is a foundational concept for effective behavior change.^34,35^ Second, every Monday the participant received 70 points (10 for each day of the week) to leverage the fresh start effect, which is the tendency for aspirational behavior around temporal landmarks such as the beginning of the year, month, or week.^36^ Participants benefited from immediate gratification each day that they achieved their step goal and, if they did not achieve their step goal, the following day they lost 10 points from their balance. This leverages prospect theory,^37^ which asserts that loss aversion provides more sustainable motivation for behavior change than gain-framing.^31,37^ Third, at the end of each week, participants could move up a level (from lowest to highest: blue, bronze, silver, gold, and platinum) if they retained 40 points or move down levels if they did not. This design creates achievable goal gradients (the notion that the next highest level is attainable), a sense of status with accomplishment, and progression through the game. Fourth, participants started at the silver level so they could experience either the accomplishment of rising to gold or the loss of dropping to bronze upon completing the first week of the intervention.

### Primary Outcome and Other Measures

The primary outcome was change in mean daily steps from baseline to the end of the 12-week intervention period. Secondary outcomes included the proportion of participants who experienced incident functional decline or mobility disability (postdischarge decrease from baseline ability to perform activities of daily living [ADLs]^38^ or ambulate without difficulty^39^) and the proportion who utilized urgent care (ie, emergency department [ED] visits and hospital readmissions) after discharge from their index hospitalization during the study period. Each ADL was scored as 1 point denoting independence, 2 points denoting difficulty, or 3 points denoting dependence in 5 domains (eating, dressing, bathing, transferring, and toileting) and scores were summed (range, 5-15, with higher scores indicating more difficulty and dependence). Mobility was scored as 0 for difficulty or 1 for no difficulty walking one-quarter of a mile. ED visits and readmissions were treated as binary (yes or no) at 30, 60, and 90 days, with data collected only from the same hospital where the patient enrolled (Hospital of the University of Pennsylvania). We performed post hoc subgroup analyses described in the next subsection using the Lubben Social Network Scale,^40^ which assesses the size of an individual’s social network (family, friends, and neighbors) and their level of engagement with that network.

### Statistical Analysis

Previous studies^13,41^ have suggested that a small difference in daily mobility (500-1500 steps per day) can be associated with clinical outcomes, including function, readmission, and mortality. A priori power calculations estimated that a sample of 300 participants (150 per group) would provide 90% power to detect a difference of 700 steps in the change in mean daily step count between intervention and control, using a 2-sided α = .05. This assumed a baseline mean (SD) step count of 5000 (2500) steps in the control group and a 10% dropout rate. Enrollment was closed at 232 participants, however, because of funding restraints on the timeline. On the basis of these same assumptions, we had 80% power to detect a 700-step difference.

One participant was randomized to the intervention but was not eligible because they did not complete the baseline period and thus was excluded from the analysis. All other randomized participants were included in the intention-to-treat analysis. For each participant on each day of the study (participant-day level), the number of steps achieved was obtained as a continuous variable. Data can be missing for any day if the participant did not use the wearable device or did not upload data; missingness in this study (34% for intervention and 31% for control) was similar to that in previous studies of physical activity interventions.^21,22,28,31^ For the prespecified main analysis, we used multiple imputation for step values that were either missing or for values less than or equal to 1000 steps per day (11% in both intervention and control). We have used this method in prior work^21,22,28,31^ because evidence indicates that daily step values less than or equal to 1000 may not represent full data capture.^42,43^ Five imputations were conducted using the mice package in R statistical software version 3.4.0 (R Project for Statistical Computing), which allows for participant random effects with this data structure.^44^ The following factors associated with missing data were included: calendar month, baseline steps, age, sex, race, education, marital status, income, comorbidities, and hospital unit. Results were combined using Rubin standard rules.^45^ This imputation approach has been used in our prior work.^21,22,28^ Sensitivity analyses were conducted using collected data without multiple imputation, both with and without step values less than or equal to 1000.

Similar to prior work,^21,22,28^ adjusted analyses used PROC GLIMMIX in SAS statistical software version 9.4 (SAS Institute) to fit linear mixed effects models with a random intercept and participant random effects and to account for the repeated measures of daily step counts. In the main adjusted model, we included baseline step count and fixed effects for calendar month and study group. We assumed a normal distribution and obtained difference in steps between groups for the intervention and follow-up periods using the least squared means command.

Finally, we performed 4 exploratory, post hoc subgroup analyses that were informed by study composition and experience and using the aforementioned methods. First, given the diverse demographic composition of our sample, we explored subgroups by race, sex, and age. Second, given our experience with patient recruitment, in which some participants had more difficulty than others naming a support partner, we explored levels of social engagement. In the Results section, we report the findings for subgroup analyses of 76 participants with higher social engagement (ie, the top one-third of scores in the Lubben Social Network Scale) and 54 middle-aged participants (50-64 years old). Analyses by race and sex were negative and thus not reported in the Results section. Data analysis was performed from October 2019 to March 2020.

## Results

We randomized 232 participants into the study (118 randomized to control and 114 randomized to the intervention), which was diverse and well-balanced between groups (Table 1). The mean (SD) age was 40 (14) years, 141 participants (61%) were women, 101 (43%) were White, 153 (66%) had some education beyond high school, 103 (44%) had an annual household income less than $50 000, 123 (53%) were single or not married, and the mean (SD) length of stay in the hospital was 4.9 (5.0) days (median [interquartile range] length of stay, 3.8 [2.7-5.6] days overall, 3.9 [2.9-6.0] days for the intervention group, and 3.7 [2.5-5.3] days for the control group). The baseline step counts in the control and intervention groups were not significantly different (3951 vs 3795 steps), and 224 participants (97%) completed the entire study. Four participants reported adverse events (2 in each group) and 1 reported a fall; no serious adverse events were reported (eTable 2 in Supplement 2).

**Table 1.** Participant Demographic and Clinical Characteristics

Characteristic	Participants, No. (%)	
	Control (n = 118)	Intervention (n = 114)
Sociodemographic		
Age, mean (SD), y	40.4 (14)	39.7 (15)
Sex		
Female	73 (62)	68 (60)
Male	45 (38)	46 (40)
Race/ethnicity		
Non-Hispanic		
White	60 (51)	41 (36)
Black	47 (40)	56 (49)
Asian	4 (3.4)	5 (4.4)
Hispanic	3 (2.5)	9 (7.9)
Other^a^	4 (3.4)	3 (2.6)
Education		
Some high school	3 (2.5)	4 (3.5)
High school graduate	30 (25)	42 (37)
Some college or specialized training	44 (37)	37 (33)
College graduate	41 (35)	31 (27)
Marital status		
Single	62 (53)	61 (54)
Married	40 (34)	41 (36)
Other	16 (14)	12 (11)
Annual household income, $		
<50 000	47 (40)	56 (49)
50 000-100 000	42 (36)	36 (32)
>100 000	29 (25)	22 (19)
Clinical		
Charlson Comorbidity Index score, median (IQR)	2 (0-4)	2 (1-4)
Discharge diagnosis		
Infectious disease	29 (13)	27 (12)
Gastrointestinal	18 (8)	20 (9)
Pulmonary	14 (6)	19 (8)
Cardiovascular	17 (7)	10 (4)
Hematologic	11 (5)	8 (3)
Renal or electrolytes	6 (3)	7 (3)
Oncologic	8 (3)	5 (2)
Other (eg, dermatologic, urologic, or toxicology)	4 (2)	7 (3)
Psychiatric	3 (1)	5 (2)
Endocrine	4 (2)	2 (1)
Rheumatologic	2 (1)	3 (1)
Neurologic	3 (1)	0
Hospital unit		
General medicine	104 (88)	105 (92)
Oncology	14 (12)	9 (8)
Inpatient length of stay, mean (SD), d^b^	4.8 (5.0)	5.1 (5.0)
Self-reported measures, mean (SD)		
Short Form-12		
Physical component	35.3 (11)	32.6 (10)
Mental component	45.7 (10)	46.6 (10)
Baseline measures, mean (SD)		
Baseline step count, No.	3951 (2586)	3795 (2824)
Body mass index^c^	29.7 (9)	26.1 (8)

Abbreviation: IQR, interquartile range.

^a^ Other includes Native American or Alaska Native and Native Hawaiian or other Pacific Islander.

^b^ The median (IQR) length of stay was 3.9 (2.9-6.0) days for the intervention group, and 3.7 (2.5-5.3) days for the control group.

^c^ Body mass index is calculated as weight in kilograms divided by height in meters squared.

### Main Analyses

**Figure 2.**
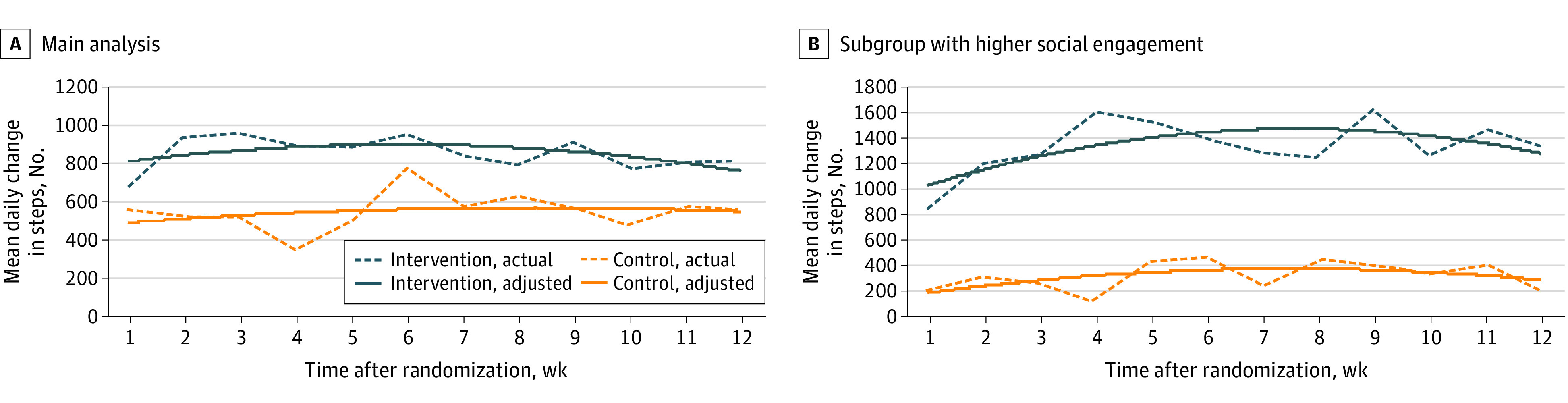
Unadjusted Increase in Mean Daily Steps From Baseline Depicted are the changes in mean daily steps for each group by week after enrollment using imputed data. Dashed lines represent actual values and solid lines represent adjusted values. Panel A shows findings for the main analysis (118 participants in the control group and 114 participants in the intervention group), and panel B shows findings for the subgroup with higher social engagement (40 participants in the control group and 36 participants in the intervention group).

Overall analysis (Table 2 and Figure 2A) showed that daily step counts increased from 3795 to 4652 steps (difference, 857 steps; 95% CI, 488 to 1224 steps) among intervention participants and increased from 3951 to 4499 steps (difference, 548 steps; 95% CI, 193 to 903 steps) among control participants (absolute difference between groups, 304 steps). The mean increase in daily steps from baseline for intervention participants was not significantly different than that for control participants (adjusted difference, 270 steps; 95% CI, −214 to 754 steps; *P* = .27). Analyses using the same model with raw step data (not imputed) and including days with fewer than 1000 steps yielded similar results (Table 2).

**Table 2.** Adjusted Differences in Daily Steps Between Study Groups

Group	Baseline steps/d, No.		Intervention period, steps/d, No.		*P* value
	Control	Intervention	Control	Intervention	
Overall (n = 232)					
Participants, No.	118	114	118	114	
Daily steps, mean (SD), No.	3951 (2586)	3795 (2824)	4499 (3610)	4652 (3812)	
Difference vs control (95% CI)					
Main adjusted model^a^	NA	NA	NA	270 (−214 to 754)	.27
Model with raw data including step counts <1000/d (95% CI)	NA	NA	NA	206 (−321 to 733)	.44
Higher social engagement subgroup (n = 76)					
Participants, No.	40	36	40	36	
Daily steps, mean (SD), No.	3476 (2359)	3866 (2830)	3800 (2165)	5218 (2832)	
Difference vs control (95% CI)					
Main adjusted model^a^	NA	NA	NA	1125 (409 to 1841)	.002
Model with raw data including step counts <1000/d (95% CI)	NA	NA	NA	1026 (345 to 1707)	.003
Age 50-64 y subgroup (n = 54)					
Participants, No.	29	26	29	26	
Daily steps, mean (SD), No.	3407 (2472)	2561 (1372)	3278 (2555)	3417 (1892)	
Difference vs control (95% CI)					
Main adjusted model^a^	NA	NA	NA	463 (−267 to 1193)	.21
Model with raw data including step counts <1000/d (95% CI)	NA	NA	NA	552 (−233 to 1337)	.17

Abbreviation: NA, not applicable.

^a^ Both main model and model with steps fewer than 1000 per day adjust for baseline step count, repeated measures, and has fixed effects for calendar month and study group.

Overall, participants in this study had high function at baseline (mean [SD] ADL score, 5.5 [1.3] overall, 5.6 [1.0] in the intervention group, and 5.4 [1.0] in the control group) on a scale of 5 to 15, where 5 is completely independent and 6 indicates difficulty in 1 ADL. One week after discharge, this mean (SD) score increased to 5.6 (1.0) (indicating functional decline) with no significant differences by group. Analysis of function as a binary outcome (incident functional disability) at 30, 60, and 90 days after discharge was also nonsignificant in the main analysis, as was analysis of mobility as a binary outcome (incident mobility disability) and urgent care utilization (ED visits or hospital readmissions) at the same time points. Response rates for function and mobility ranged from were 67% to 80% across all time points.

### Subgroup Analyses

Results of post hoc analysis of the primary outcome for 76 participants with higher levels of social engagement (top one-third of scores in the Lubben Social Network Scale) is shown in Table 2 and Figure 2B. Intervention participants in this subgroup had a significant increase in mean daily steps from baseline compared with control participants who also had higher levels of social engagement (adjusted difference, 1125 steps; 95% CI, 409 to 1841 steps; *P* = .002). We also conducted subgroup analysis of middle-aged participants (aged 50-64 years). Intervention participants in this subgroup had an increase from baseline steps compared with control participants that was larger than that in the main analysis but the difference was not significant (adjusted difference, 463 steps; 95% CI, −267 to 1193 steps; *P* = .21). Results were similar in sensitivity analyses that used collected data without multiple imputation for both the main and subgroup analyses (eTable 3 and eTable 4 in Supplement 2).

**Figure 3.**
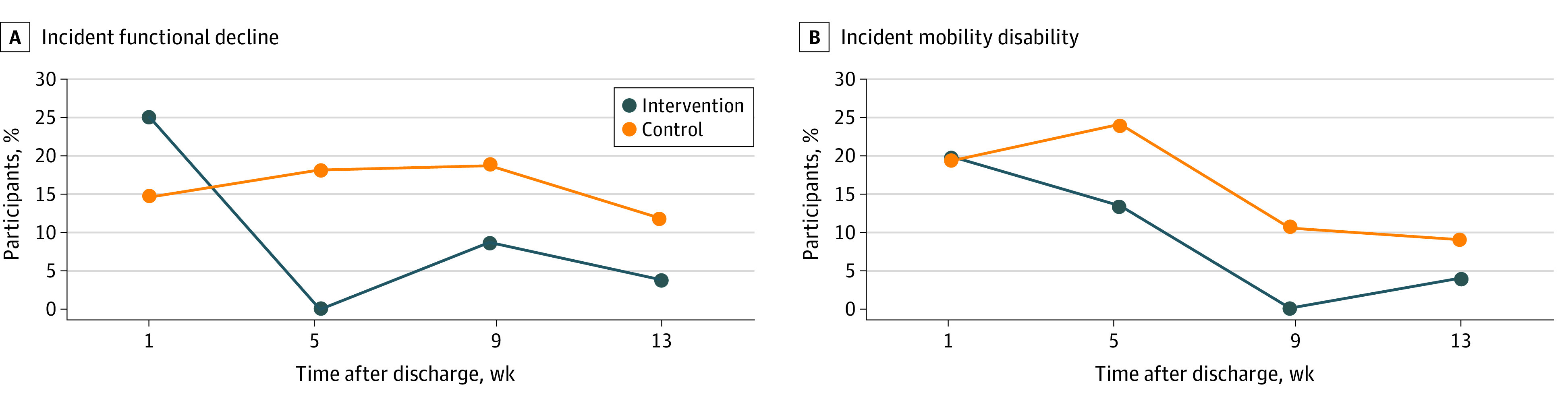
Incident Functional Decline and Mobility Disability in Subgroup Analysis Panel A depicts new functional disability (increased difficulty with activities of daily living from baseline), and panel B depicts new mobility disability (increased difficulty walking a quarter mile) by study group beginning 1 week after discharge and ending after 12 subsequent weeks for the intervention.

With respect to secondary outcomes, in post hoc analyses for participants with higher social engagement, the proportion of participants with incident functional disability was lower in the intervention group than in the control group at all time points, and fewer intervention participants experienced persistent functional decline at the end of the study (Figure 3A). Although the mean change in ADL score from baseline was small in the intervention group, it was significantly different from that in the control group (mean score change, 0.18; 95% CI, 0.002-0.35; *P* = .047). Similarly, in assessing mobility disability as a binary outcome for all participants, incident mobility disability was less common among intervention participants than control participants at all time points and fewer participants experienced persistent mobility decline at the end of the study (Figure 3B). Intervention participants had lower unadjusted odds of persistent mobility disability at week 13 (odds ratio, 0.11; 95% CI, 0.01-0.92; *P* = .04). Finally, there was no difference in urgent care utilization (ED visits and hospital readmissions after discharge from the index hospitalization) in subgroup analyses at 90 days. There was a reduction in readmissions in the subgroup with higher social engagement at 30 days but the difference was not significant (3 of 36 readmissions [8%] for the intervention group vs 6 of 40 readmissions [15%] for the control group). Participants in this subgroup also had less functional decline (1 of 36 participants [4%] in the intervention group vs 5 of 40 participants [12%] in the control group) but the differences were not significant.

## Discussion

This randomized clinical trial testing the effects of a gamification intervention with social incentives to improve mobility after hospitalization did not find a significant increase in step counts for the overall study. Previous trials^21,22,28,31^ by our group using behaviorally designed gamification with social incentives in community settings have successfully increased mobility from 6000 to 7000 steps per day at baseline to 8000 to 9000 steps per day, with the goal to ultimately reduce 10-year cardiovascular risk. In the present study, we applied this approach to a high-risk population of hospitalized patients with a much lower baseline (3000-4000 steps per day) with a novel goal of reducing short-term functional decline and urgent care utilization. On the basis of observational studies, we have hypothesized a mobility-toxicity curve^46^ for hospitalized patients; to our knowledge, the present study is one of the first interventional studies to test the effects of a small increase in steps to avoid these outcomes. In addition, results of post hoc exploratory analyses with patients who had higher levels of social engagement provide critical direction for future studies using behavioral economics to improve mobility and function in this population. There are several factors that may explain why this novel application of robust, behaviorally designed gamification with social incentives were not effective in our main analysis.

First, with respect to our primary outcome of mobility, lower levels of social engagement in this population may have hindered the impact of the social incentive intervention component. Similar to prior studies, the social incentive used in this study relied on participant engagement with a support partner; however, prior studies have recruited populations with higher levels of social engagement. The BE FIT^21^ trial recruited only individuals living within families, and STEP UP^22^ recruited only individuals working for a national consulting firm. Many participants in the current study reported very little support from family, workplace, or community; some were unable to name 1 person whom they felt could be relied on to receive information on the participant’s progress and send messages for reinforcement and encouragement to the participant. Exploratory subgroup analysis of participants with higher social engagement showing a significant increase in mobility (>1000 more steps per day than the control group) seems to support our initial hypothesis that social incentives can work in a hospitalized population, but with an important caveat that they must have the social network to enable this mechanism. Future studies should explore mechanisms to improve the social support available with a particular focus on populations at high risk for social isolation, such as older adults. This could include peer-level engagement with health coaches or community health workers^47,48^ who can meet these patients where they live and provide more direct support to increase mobility.

Second, with respect to our secondary outcome of functional decline, broad enrollment criteria intended to maximize generalizability may have limited intervention effects. The young age and high functional baseline for participants who enrolled in this study almost certainly limited our ability to detect changes in functional status after discharge. On the other hand, several recent studies have shown that functional decline is more common in middle age than generally recognized^49,50^ and is specifically more common in those who are hospitalized.^16,51^ Moreover, this is the first study, to our knowledge, to test the hypothesis that increased mobility (steps per day) after hospitalization can prevent functional decline using a robust randomized trial design. Although we did not find evidence to conclude that higher mobility prevents functional decline in all participants, we did find a promising signal in post hoc subgroup analyses with those who had higher social engagement. With respect to older subgroups, we observed greater mobility and less functional decline for middle-aged (aged 50-64 years) but not older (aged ≥65 years) subgroups, although the differences were not significant; however, both of these analyses were underpowered because of the small sample sizes. This contributes to a broader discussion about whether posthospital functional decline affects older vs middle-aged groups differently.^16,51^ We believe our pilot study of behaviorally designed gamification with social incentives provides a signal for future studies to improve posthospital function by focusing on an older, frailer population at higher risk for impaired mobility and functional decline.^1,2,15,16^

### Strengths and Limitations

This study has strengths and limitations that should be considered when interpreting our findings. Strengths include the application of a robust gamification and social support approach to a novel population of recently hospitalized patients and program automation, which limits the need for personnel costs and enables scalability. The apparent effects among participants with higher social engagement also provide opportunities to tailor this intervention and target higher-risk populations such as middle-aged and older adults. This study also has several limitations. First, participants were recruited from a single urban academic medical center and were younger and lower socioeconomic status than national averages for patients admitted to general medicine and oncology units, which may limit generalizability of the findings. Our inclusion criteria of ability to ambulate independently (Activity Measure for Post-Acute Care score ≥12) may have unintentionally excluded older adults who required some assistance to ambulate during hospitalization. These interventions should be tested in other populations and settings, particularly among older adults in community hospital settings. Second, we evaluated mobility using patient-generated step counts and patient-reported functional status without in-person evaluation of mobility or function (eg, timed up-and-go test). Future studies could include in-person assessments of mobility and function, particularly in older, functionally high-risk populations. Third, our intervention did not start until after participants had been discharged from the hospital. Future studies should evaluate interventions to increase mobility that begin during hospitalization and extend into the postdischarge period. Fourth, we do not have data on how often support partners engaged with participants to encourage them, although interviews with a small sample of participants suggest that social support was perceived to facilitate success in achieving mobility goals. Fifth, although a 12-week intervention is longer than many postdischarge interventions that focus on the first 30 days, future studies should strive to enable sustainability over longer periods.

## Conclusions

This 12-week randomized clinical trial of behaviorally designed gamification with a support partner in adult, hospitalized patients did not increase postdischarge mobility (steps per day) or decrease functional decline compared with the control group for the overall sample. Significant findings in post hoc subgroup analyses suggest that this intervention may be effective in some populations, such as those with higher social engagement. Future applications of this approach may need to combine other forms of social support or target older adults at higher risk to impact outcomes of mobility and function after discharge from general medicine and oncology units.

## Notes

Supplement 1. **Trial Protocol**

Supplement 2. **eTable 1. **Validated Instruments for Patient-Reported Outcomes in MOVE IT Study **eReferences.** **eTable 2.** Adverse Events Reported **eTable 3.** Adjusted Differences in Daily Steps Using Collected Data (No Imputations) **eTable 4.** Adjusted Differences in Daily Steps Using Collected Data (No Imputations) and Excluding Step Values < 1000

Supplement 3. **Data Sharing Statement**
