# Supplementary material for: Effect of Behaviorally Designed Gamification With a Social Support Partner to Increase Mobility After Hospital Discharge: A Randomized Clinical Trial
Source: JAMA Netw Open. 2021 Mar 24;4(3):e210952. doi: 10.1001/jamanetworkopen.2021.0952 (PMC7991973; doi:10.1001/jamanetworkopen.2021.0952)
Supplement: Supplement 1. — Trial Protocol [file jamanetwopen-e210952-s001.pdf]

Supplement 1

Original Protocol

Social Incentives to Increase Mobility Among Hospitalized Patients: The MOVE IT Randomized Clinical Trial

Study Protocol

November 29, 2017

Note: There were no intervention protocol changes during the study.

Outline

1. Abstract
2. Overall objectives
3. Aims
    - 3.1 Primary outcome
    - 3.2 Secondary outcomes
4. Background
5. Study design
    - 5.1 Design
    - 5.2 Study duration
    - 5.3 Target population
    - 5.4 Accrual
    - 5.5 Key inclusion criteria
    - 5.6 Key exclusion criteria
6. Subject recruitment
    - 6.1 General
    - 6.2 Populations vulnerable to undue influence or coercion
7. Subject compensation
8. Study procedures
    - 8.1 Consent
    - 8.2 Procedures
9. Analysis plan
10. Investigators
11. Human research protection
    - 11.1 Data confidentiality

11.2 Subject confidentiality

11.3 Subject privacy

11.4 Data disclosure

11.5 Data safety and monitoring

11.6 Risk/benefit

11.6.1 Potential study risks

11.6.2 Potential study benefits

11.6.3 Risk/benefit assessment

11.6.4 Alternatives to participation

1. Abstract

Hospital-associated functional decline is a common, costly, and important event in the health trajectory of older adults. Mobility has been identified as a potential mediator for many of these outcomes. In this study, we will use wearable devices to monitor patients’ physical activity during inpatient admission on floors with and without nursing mobility protocols that provide guidance on recommended activity. This is part of a health system initiative to deploy a hospital-based mobility protocol across the entire system over time. Upon discharge, we will measure each patient’s baseline level of physical activity and then randomly assign them to 3 months of passive monitoring or a social incentive-based gamification intervention with personalized step goals to increase activity. We will monitor patient outcomes and conduct regular validated surveys on patient function.

2. Overall objectives

The primary objective is to assess the effectiveness of a social incentive-based gamification intervention to increase physical activity in the 3 months after hospital discharge. We will explore patients’ physical activity while in the hospital and if that differs across floors that have or have not deployed a nursing mobility protocol. We will also explore changes in patient functional decline and 30-day hospital readmission.

3. Aims

3.1 Primary outcome

The primary outcome variable is the change in mean daily step count from the baseline period (week 1 post-discharge) to the intervention period (weeks 2-13 post-discharge).

3.2 Secondary outcome

The secondary outcome variables are functional decline from admission to 3-months post-discharge and 30-day hospital readmission.

4. Background

Hospitalization is a common occurrence for older adults; approximately 6.8 million Medicare seniors experience an admission for acute care in any given year.(1,2,3) This is often a sentinel event in the overall health trajectory of older adults that is complicated by functional impairment,(4,5) Skilled Nursing Facility placement, (6,7) and reduced mobility after discharge.(8)

In the current paradigm, low mobility during hospitalization is largely viewed as a temporary inconvenience that should not affect overall functional ability or outcomes such nursing home placement and that patients should return to their previous activity level soon after they return home without lingering mobility changes. Recent research, however, suggests disruptions of basic activities of daily life such as mobility (getting out of bed and walking) may be

"traumatic"(9) or "toxic"(10) to older adults with long-term post-hospital effects.(11) What we lack is precise data on how much immobility is noxious and how much mobility is needed to protect against adverse outcomes.

1. Gorina Y, Pratt LA, Kramarow EA, Egaddal N. Hospitalization, Readmission, and Death Experience of Noninstitutionalized Medicare Fee-for-service Beneficiaries Aged 65 and Over. CDC National Health Statistics Reports. Number 84, Sept 28, 2015

2. AARP Public Policy Institute. The Medicare Beneficiary Population Fact Sheet. Accessed online on February 17, 2016 at: http://assets.aarp.org/rgcenter/health/fs149_medicare.pdf

3. The Dartmouth Atlas of Health Care. Hospital Discharges per 1,000 Medicare enrollees. Accessed online on February 17, 2016 at: http://www.dartmouthatlas.org/data/topic/topic.aspx?cat=19

4. Covinsky KE, Pierluissi E, Johnston CB. Hospitalization-associated disability: "She was probably able to ambulate, but I'm not sure". JAMA. 2011 Oct 26;306(16):1782-93.

5. Covinsky KE, Palmer RM, Fortinsky RH, et al. Loss of independence in activities of daily living in older adults hospitalized with medical illnesses: increased vulnerability with age. J Am Geriatr Soc. Apr 2003;51(4):451-458

6. Brock AM, OSullivan P. A study to determine what variables predict institutionalization of elderly people. Journal of Advanced Nursing, 1985; 10: 533537.

7. Fortinsky RH, Covinsky KE, Palmer RM, Landefeld CS. Effects of functional status changes before and during hospitalization on nursing home admission of older adults. J Gerontol A Biol Sci Med Sci. 1999;54(10):M521-M526.

8. Brown CJ, Roth DL, Allman RM, Sawyer P, Ritchie CS, Roseman JM. Trajectories of Life-Space Mobility after Hospitalization. Ann Intern Med 150(6):372-378, 2009.

9. Detsky AS, Krumholz HM. Reducing the trauma of hospitalization. JAMA. 2014 Jun 4;311(21):2169-70.

10. Creditor MC. Hazards of hospitalization of the elderly. Ann Intern Med. 1993 Feb 1;118(3):219-23.

11. Krumholz HM. Post-hospital syndrome - An acquired, transient condition of generalized risk. N Engl J Med 2013; 368:100-102**5.**

Study design

5.1 Design

We will conduct a two-arm randomized, controlled trial during the 3-months after hospital discharge comparing a control group that uses a wearable device to track physical activity to an intervention group that uses the same wearable devices and receives a supportive social incentive-based gamification intervention to adhere to a step goal program. Patients will be enrolled during hospitalization from medicine and oncology floors into three phases. In phase 1 (hospitalization), patients' inpatient step counts will be monitored. In phase 2 (week 1 post-discharge), patients will have a baseline step count estimated. In phase 3 (weeks 2-13 post-discharge), patients will be randomly assigned to the control or intervention group. Patients will be considered enrolled in the trial if they complete the run-in periods (phases 1 and 2) and then are randomized into phase 3.

The enrollment phase will be part of another randomized trial (The RETAIN study) which evaluates how the magnitude of financial incentives impacts patient enrollment. This trial will be completed and unmasked to patients before they begin the phase 1 of our study.

5.2 Study duration

The study will begin in Winter 2018. The primary intervention period is the 3-months post-hospital discharge. We expect the entire study to take approximately two years to conduct and analyze outcomes.

5.3 Target population

Adults age 18 years or older who are admitted for inpatient care to the Hospital of the University of Pennsylvania (HUP) on medicine or oncology floors.

5.4 Accrual

We will aim to enroll and randomize 300 patients. We estimate that a sample size of at least 300 participants (150 per arm) will provide 90% power to detect a difference of 700 steps in the change in mean daily step count from baseline to the intervention period between intervention and control, using a 2-sided α of 0.05, assuming a baseline mean step count of 5000 steps in the control group with a standard deviation of 2500 steps, and accounting for a 10% dropout rate.

5.5 Key inclusion criteria

1) Age 18 years or older; 2) admitted to medicine or oncology floor in the hospital

5.6 Key exclusion criteria

1) Inability to provide informed consent; 2) does not have daily access to a smartphone compatible with the wearable device and not willing to use a device that we can provide them; 3) already enrolled in another physical activity study; 4) inpatient mobility score of 0 or 1 indicating that physical activity is not appropriate for the patient; 5) any other medical conditions that would prohibit participation in an 3-month physical activity program; 6) unable to complete the run-in phases (e.g. not discharged from the hospital within 60 days of enrolling in the study; not willing to use the wearable device for the 3-month post-discharge intervention)

6. Subject recruitment

6.1 General

The study team will use the electronic health record to identify potentially eligible patients in the hospital. The enrollment portion of the study will be conducted using the protocol from the RETAIN study.

6.2 Populations vulnerable to undue influence or coercion

Not applicable

7. Subject compensation

Participants will receive $150 for enrolling and $150 for completing the 3-month post-discharge period.

8. Study procedures

8.1 Consent

Using a REDCap survey module on a tablet, the clinical research coordinator will read a pre-MOVE IT consent script (see Script 2_Pre-MOVE IT Consent Intro Script) and proceed to present the MOVE IT informed consent/ HIPAA forms with additional information regarding the RETAIN incentive randomization condition. Participants will be able to progress through the consent form at their own pace, as well as review the consent form on one full page at the end on the tablet. REDCap will capture the time spent reading the individual sections of the consent form, which will be featured as images.

Once participants finish reading the electronic version of the MOVE IT consent form with randomized compensation information, they will hand the tablet back to the clinical research coordinator.

The clinical research coordinator will then toggle back to the Way to Health in order to present the next set of surveys. Patients will then complete brief assessments of perceptions of research risk, perceptions of the difference between research and individualized patient care, and understanding of the MOVE IT study via a trial elements quiz (please see Attachments RETAIN_1 through RETAIN_9 surveys). Patients' advancement through the consent process

will not be contingent on their responses to the quiz; however, following completion of the quiz, research coordinators will review the questions that patients missed and discuss these portions of the consent until the participant understands the entire consent and its detailed procedures.

Participants will be provided with the paper consent form for signature and will be asked to either provide consent or decline participation in the parent trial (MOVE IT). In the paper copy of the consent, details regarding how to contact the research team via email or phone will be provided in the event a participant wants to withdraw from the study.

As part of the collaboration with RETAIN, we seek approval to alter the required elements of the informed consent process in order to temporarily withhold information regarding the randomization to one of three incentives for research participation. All patients who consent to MOVE IT will be debriefed in the same encounter during which they were approached. Please see Script_3_Debriefing Script_RETAIN_MOVE IT, which includes the following information:

- Clinical research coordinator will describe the study's use of deception and purpose.
- Patients will also be informed of payment equalization (e.g. all participants will receive a $300 incentive amount in two payments.
- Patients will be asked if they would like to receive their payment.
- In order to receive their payment of $300 in two installments ($150 to be processed immediately and $150 during once their 3-month follow-up for MOVE IT is complete), participants may be asked to complete a W-9.

8.2 Procedures

Each day, the research coordinator will use an electronic health record query to generate a list of patients that are admitted to medicine and oncology floors and meet AMPAC and age requirements. At enrollment, the research coordinator will administer two additional screening questions to determine eligibility (owns a smart phone and not in another physical activity study) via the RedCap portal. If the participant successfully passes the screening questions, the patient can be consented to the study. The research coordinator will next present a series of to the patient to be completed through the Way to Health portal. These instruments include: the Pittsburgh Sleep Quality Index, Karolinska Scale, Activity of Daily Living-Instrumental Activities of Daily Living Survey, Life Space Activity Survey, Mini-Nutritional Assessment Survey, Edmonton Symptom Assessment Survey, Treatment Burden Questionnaire, Lubbens Social Network Scale, Self Rated Health Survey, and Dementia Tool.

After completing the enrollment assessment, the research coordinator will show patients how to use the wearable device which can be worn on wrist (like a watch), carried in any pocket, or clipped to hospital gown. After they are enrolled, participants will receive a daily text message

with a link to complete two surveys in Way to Health about their sleep quality and symptom assessment for up to 10 days of hospital admission, or until they are discharged.

Upon hospital discharge, there will be a one-week run-in period after discharge to estimate baseline step counts for all patients. After week 1, all participants will be notified to complete post-discharge baseline surveys. For all patients with a baseline step count, they will be randomly assigned to control or intervention using block sizes of two and stratifying on baseline step count (<5000 daily steps, 5001 to 7500, or > 7500) and hospital admission floor.

Participants in the control group will continue to have data collected passively. Participants in the control arm will only receive study communications when it is time for them to complete surveys in the Way to Health system.

Participants in the intervention arm will have a weekly step goal that increases from baseline by 10% each week of the intervention (12 weeks) with a maximum of 10,000 steps. At the beginning of each week, the participant receives 70 points (10 points for each day that week). If the participant does not meet their step goal, they lose 10 points from their balance. This leverages loss aversion, which has been demonstrated to motivate behavior change more effectively with losses than gains. At the end of each week if the participant has at least 40 points, they will move up a level (levels from lowest to highest: blue, bronze, silver, gold, platinum). If not, the participant will drop a level. All participants begin at the silver level. Each week, participants get a fresh set of 70 points on Monday. Participants will receive daily feedback for the step counts, and weekly feedback for their levels. Participants in the intervention arm will be asked to identify a family member or friend to be their support sponsor. A weekly report will be sent to this person with the participant’s performance (points and level).

Participants in both arms will also receive a notification when it is time to complete surveys in the Way to Health system (5, 9, and 13 weeks after hospital discharge). 3 days after the surveys are sent to participants, a message will be sent reminding them to complete the surveys if they have not already done so. Upon completion of the final set of surveys, $150 will be processed for participants in both arms.

9. Analysis plan

In our primary analyses, we will multiple imputation for missing data and use linear mixed effects models to compare the change in mean daily step count from baseline to 12-week intervention period, adjusting for baseline step count and time. To test of the robustness of our findings we will also evaluate models using collected data without imputation. All hypothesis tests will be two-sided using a two-sided alpha of 0.05 as our threshold for statistical significance. In secondary analyses, we will evaluate how differences in inpatient and changes in post-discharge step counts are associated with secondary outcome measures.

In exploratory analyses, we will compare how differences in hospital mobility protocols are associated with differences in step counts. We will also conduct a qualitative analysis to evaluate participant perceptions of the intervention.

10. Investigators

Ryan Greysen, MD, MHS, MA is the Principle Investigator (PI). He is Chief of the Section of Hospital Medicine in the Division of General Internal Medicine and an Assistant Professor of Medicine at the Perelman School of Medicine at the University of Pennsylvania. He has past experience leading studies of older, hospitalized adults focused on functional vulnerability and outcomes including hospital readmission. He currently spends 75% of his effort on research and 25% on clinical and administrative activities.

Mitesh Patel, MD, MBA, MS (Co-Investigator) is an Assistant Professor of Medicine and Health Care Management at the Perelman School of Medicine and The Wharton School at the University of Pennsylvania. He has past experience leading six clinical trials using the Way to Health Platform to deploy interventions using financial and social incentives to promote weight loss and increased physical activity. He currently spends 80% of his effort on research and 20% on clinical and teaching activities.

Heather Greysen (Co-Investigator) is a Fellow in the New Courtland Center for Transitions in Care in the School of Medicine at the University of Pennsylvania. She has previously led projects to understand the effects of physical activity on health and wellbeing of older adults with chronic disease. She currently spends 90% of her effort in clinical and teaching activities.

The Clinical Research Coordinator has experience with administering studies involving behavioral interventions and financial incentives, and also has experience training Research Assistants to follow study protocols.

11. Human research protection

11.1 Data confidentiality

Paper-based records will be kept in a secure location and only be accessible to personnel involved in the study. Computer-based files will only be made available to personnel involved in the study through the use of access privileges and passwords. Wherever feasible, identifiers will be removed from study-related information. Precautions are in place to ensure the data are secure by using passwords and encryption, because the research involves web-based surveys.

11.2 Subject confidentiality

Research material will be obtained from participant surveys, from the wearable devices, and from the electronic health record. All participants will provide informed consent for access to

these materials. The data to be collected include demographic data (e.g., age, sex, self-identified race), outcome data, and daily activity data collected by the wearable device. Research material that is obtained will be used for research purposes only. The same procedure used for the analysis of automated data sources to ensure protection of patient information will be used for the survey data, in that patient identifiers will be used only for linkage purposes or to contact patients. The study identification number, and not other identifying information, will be used on all data collection instruments. All study staff will be reminded to appreciate the confidential nature of the data collected and contained in these databases. The Penn Medicine Academic Computing Services (PMACS) will be the hub for the hardware and database infrastructure that will support the project and is where the Way to Health web portal is based. The PMACS is a joint effort of the University of Pennsylvania's Abramson Cancer Center, the Cardiovascular Institute, the Department of Pathology, and the Leonard Davis Institute. The PMACS provides a secure computing environment for a large volume of highly sensitive data, including clinical, genetic, socioeconomic, and financial information. Among the IT projects currently managed by PMACS are: (1) the capture and organization of complex, longitudinal clinical data via web and clinical applications portals from cancer patients enrolled in clinical trials; (2) the integration of genetic array databases and clinical data obtained from patients with cardiovascular disease; (3) computational biology and cytometry database management and analyses; (4) economic and health policy research using Medicare claims from over 40 million Medicare beneficiaries. PMACS requires all users of data or applications on PMACS servers to complete a PMACS-hosted cybersecurity awareness course annually, which stresses federal data security policies under data use agreements with the university. The curriculum includes Health Insurance Portability and Accountability Act (HIPAA) training and covers secure data transfer, passwords, computer security habits and knowledge of what constitutes misuse or inappropriate use of the server. We will implement multiple, redundant protective measures to guarantee the privacy and security of the participant data. All investigators and research staff with direct access to the identifiable data will be required to undergo annual responsible conduct of research, cybersecurity, and HIPAA certification in accordance with University of Pennsylvania regulations.

Data will be stored, managed, and analyzed on a secure, encrypted server behind the University of Pennsylvania Health System (UPHS) firewall. This server was created for projects conducted by the Penn Medicine Nudge Unit related to physician and patient behavior at UPHS. All study personnel that will use this data are listed on the IRB application and have completed training in HIPAA standards and the CITI human subjects research. Data access will be password protected. Whenever possible, data will be de-identified for analysis.

11.3 Subject privacy

Enrollment will include a description of the voluntary nature of participation, the study procedures, risks and potential benefits in detail. The enrollment procedure will provide the opportunity for potential participants to ask questions and review the consent form information

with family or caregivers prior to making a decision to participate. Participants will be told that they do not have to answer any questions if they do not wish and can drop out of the study at any time, without affecting their medical care or the cost of their care. They will be told that they may or may not benefit directly from the study and that all information will be kept strictly confidential, except as required by law. Subjects will be given a copy of the consent document. All efforts will be made by study staff to ensure subject privacy.

11.4 Data disclosure

The following entities, besides the members of the research team, may receive protected health information (PHI) for this research study: -Wells Fargo, the company which processes study-related payments. Patient addresses and account balances will be stored on their secure computers. -Nokia, the company that designs and manufactures the wearable devices used in the study to track participant physical activity. RedCap, a secure web application where participants' informed consent forms will be stored. -Twilio, Inc., the company which processes some study-related messages. Twilio will store patients' phone numbers on their secure computers. -Qualtrics, Inc., the company which processes most study-related surveys. Qualtrics will house de-identified answers to these surveys on their secure servers. -The Office of Human Research Protections at the University of Pennsylvania -Federal and state agencies (for example, the Department of Health and Human Services, the National Institutes of Health, and/or the Office for Human Research Protections), or other domestic or foreign government bodies if required by law and/or necessary for oversight purposes.

11.5 Data safety and monitoring

At the time of enrollment in the hospital, all patients will be given anticipatory guidance on when to seek medical attention (e.g. when to call their nurse or doctor should they feel dizzy, short of breath, chest pain, lightheaded, unstable, or otherwise unwell while ambulating). In addition, participants will be asked to report to the study team any episodes of these symptoms that occur during ambulation during visits with research staff (the research assistant will check in periodically with enrolled patients to ensure devices are working properly and troubleshoot any issues encountered by patients/caregivers or nursing). Patients/caregivers will also be reminded that they can always contact the study team by phone or email at any time (contact information will be given at the beginning of the study and will also be posted on the Way to Health platform, which can be accessed at any time by the participant). If any concerns of a participant event are identified, the study coordinator will reach out to the participant and complete the event reporting form. This form will be reviewed with the study PI to determine if any action is needed and if the participant can continue safely in the study. Any identified adverse events will be reported to the Institutional Review Board. For patients on floors where the mobility protocol is in place, mobility goals are revised daily by the bedside nurse caring for the patient. If patient is noted to be weaker, the nurse will automatically reduce the mobility goal for the day per the mobility protocol (this happens without regard to research protocol).

11.6 Risk/benefit

11.6.1 Potential study risks

The major potential risk of this study is a breach of participant confidentiality. We will minimize this risk of confidentiality breach by linking individual identifying information with participant ID numbers only in one single secure file that will only be accessed by the study team in the case of an adverse medical event, participant dropout, or if otherwise deemed necessary by the Principal Investigator. All other identifying information will be discarded after initial contact with the Study Coordinator. All other members of the research team will be able to view only participant ID numbers. Additionally, participants will receive guidance from the research coordinator on when to seek medical attention (call for their nurse or doctor in the hospital) and a reporting protocol is in place to capture any changes in symptoms with physical activity.

11.6.2 Potential study benefits

Through participation in this study, each participant will have the potential to increase their physical activity, which could improve their health and reduce their risk for functional decline, falls, nursing home placement, or hospital readmission. If this approach is effective, it could have tremendous benefits for society if adopted on a wide scale to help individuals increase physical activity during hospitalization to avoid harms of hospitalization such as functional decline or nursing home placement. It is expected that other people will gain knowledge from this study and that participation could help understand how to effectively motivate people to become more physically active. Participants may also receive no benefit from their participation in the study.

11.6.3 Risk/benefit assessment

Anticipated risks of this study should be minimal and the risk/benefit ratio is very favorable. To minimize the chance for serious and unexpected adverse events, study participants will be screened through exclusion criteria. Participants that increase physical activity may improve their health and reduce their risk for hospital-associated functional decline, falls, nursing home placement, or hospital readmission.

11.6.4 Alternatives to participation

Patients do not have to be enrolled in the study in order to participate in activities to promote healthy mobility and overall patient engagement - all patients (regardless of study status) will receive usual care in these areas including physical therapy, nursing assistance with ambulation as needed, etc. Participation in the study merely entails wearing sensors to quantify mobility and answering questions from enrollment and exit surveys. Patients may receive feedback on their mobility from healthcare providers (nurses, physical therapists, doctors) without agreeing to participate in the study and wear mobility sensors.
