# Supplementary material for: Effect of Behaviorally Designed Gamification With a Social Support Partner to Increase Mobility After Hospital Discharge: A Randomized Clinical Trial
Source: JAMA Netw Open. 2021 Mar 24;4(3):e210952. doi: 10.1001/jamanetworkopen.2021.0952 (PMC7991973; doi:10.1001/jamanetworkopen.2021.0952)
Supplement: Supplement 2. — eTable 1. Validated Instruments for Patient-Reported Outcomes in MOVE IT Study eReferences. eTable 2. Adverse Events Reported eTable 3. Adjusted Differences in Daily Steps Using Collected Data (No Imputations) eTable 4. Adjusted Differences in Daily Steps Using Collected Data (No Imputations) and Excluding Step Values < 1000 [file jamanetwopen-e210952-s002.pdf]

Supplemental Online Content

Greysen SR, Changolkar S, Small DS, et al. Effect of behaviorally designed gamification with a social support partner to increase mobility after hospital discharge: a randomized clinical trial. *JAMA Netw Open*. 2021;4(3):e210952.
doi:10.1001/jamanetworkopen.2021.0952

eTable 1. Validated Instruments for Patient-Reported Outcomes in MOVE IT Study
eReferences
eTable 2. Adverse Events Reported
eTable 3. Adjusted Differences in Daily Steps Using Collected Data (No Imputations)
eTable 4. Adjusted Differences in Daily Steps Using Collected Data (No Imputations) and Excluding Step Values < 1000

This supplemental material has been provided by the authors to give readers additional information about their work.

© 2021 Greysen SR et al. *JAMA Network Open.*

eTable 1: Validated Instruments for Patient-Reported Outcomes in MOVE IT study

Concept	Survey name	Specific domain(s)	No. of questions	Frequency of administration*	Instrument validation
Function	Katz Activities of Daily Living (ADLs)	Eating, hygiene, dressing, etc.	5	1, 5, 9, 13 weeks post-discharge	[1-3]
Function	Lawton Instrumental ADLs	Taking meds, shopping, walking, etc.	15	1, 5, 9, 13 weeks post-discharge	[1-3]
Mobility	Life Space Activity	Community mobility over last 30 days	5	5, 9, 13 weeks post-discharge	[4,5]
Social Engagement	Lubben Social Network Scale	Number, frequency, and depth of social interactions	9	At enrollment only	[6]
Sleep	Pittsburgh Sleep Quality Index	Quality over last 30 days	9	5, 9, 13 weeks post-discharge	[7,8]
Sleep	Karolinska Scale	Quality of prior night	7	Daily during hospital stay only	[9,10]
Cognition	Short Portable Form	Cognitive impairment	10	At enrollment only	[11]
Self-rated Health	Short Form Health Survey (SF-12)	Physical and Cognitive domains	12	5, 9, 13 weeks post-discharge	[12,13]
Symptom Burden	Edmonton Symptom Assessment Survey	Perceived stress of pain, nausea, anxiety, etc.	8	Daily during hospital stay and 1, 5, 9, 13 weeks post-discharge	[14,15]
Treatment Burden	Treatment Burden Questionnaire	Perceived stress of tests and treatments	6	At enrollment only	[16]
Nutrition	Mini-Nutritional Assessment Survey	Nutritional status over last 30 days	6	5, 9, 13 weeks post-discharge	[17-19]

*All instruments were administered at time of enrollment

eReferences

1. Lawton MP, Brody EM. Assessment of older people: self-maintaining and instrumental activities of daily living. *The Gerontologist.* 1969;9(3):179-186.
2. Pearson V. Assessing Older Persons. Measures, Meaning and Practical Application. In: Kane R, Kane R, eds. *Assessment of Function.* New York: Oxford University Press; 2000:17-48.

© 2021 Greysen SR et al. *JAMA Network Open.*

3. Katz S, Ford AB, Moskowitz RW, et al. Studies of Illness in the Aged. The Index of ADL: A Standardized Measure of Biological and Psychosocial Function. *JAMA.* 1963;185:914-19.
4. Peel C, Sawyer Baker P, Roth DL, et al. Assessing mobility in older adults: the UAB Study of Aging Life-Space Assessment. *Phys Ther.* 2005;85(10):1008-1119.
5. Baker PS, Bodner EV, Allman RM. Measuring life-space mobility in community-dwelling older adults. *J Am Geriatrics Soc.* 2003;51(11):1610-1614.
6. Lubben J, Blozik E, Gillmann G, et al. Performance of an abbreviated version of the Lubben Social Network Scale among three European community-dwelling older adult populations. *Gerontologist.* 2006;46(4):503-513.
7. Buysse DJ, Reynolds CF, 3rd, Monk TH, et al. The Pittsburgh Sleep Quality Index: a new instrument for psychiatric practice and research. *Psychiatry research.* 1989;28(2):193-213.
8. Mollayeva T, Thurairajah P, Burton K, et al. The Pittsburgh sleep quality index as a screening tool for sleep dysfunction in clinical and non-clinical samples: A systematic review and meta-analysis. *Sleep med rev.* 2016;25:52-73.
9. Geiger Brown J, Wieroney M, Blair L, et al. Measuring subjective sleepiness at work in hospital nurses: validation of a modified delivery format of the Karolinska Sleepiness Scale. *Sleep & breathing = Schlaf & Atmung.* 2014;18(4):731-739.
10. Keklund G, Akerstedt T. Objective components of individual differences in subjective sleep quality. *J Sleep Res.* 1997;6(4):217-220.
11. Pfeiffer E. A short portable mental status questionnaire for the assessment of organic brain deficit in elderly patients. *J Am Geriatrics Soc.* 1975;23(10):433-441.
12. Resnick B, Nahm ES. Reliability and validity testing of the revised 12-item Short-Form Health Survey in older adults. *Journal Nursing Measurement.* 2001;9(2):151-161.
13. Ware J, Jr., Kosinski M, Keller SD. A 12-Item Short-Form Health Survey: construction of scales and preliminary tests of reliability and validity. *Medical Care.* 1996;34(3):220-233.
14. Bruera E, Kuehn N, Miller MJ, et al. The Edmonton Symptom Assessment System (ESAS): a simple method for the assessment of palliative care patients. *Journal Palliative Care.* 1991;7(2):6-9.
15. Richardson LA, Jones GW. A review of the reliability and validity of the Edmonton Symptom Assessment System. *Curr Oncol.* 2009;16(1):55.
16. Tran VT, Harrington M, Montori VM, Barnes C, Wicks P, Ravaud P. Adaptation and validation of the Treatment Burden Questionnaire (TBQ) in English using an internet platform. *BMC Med.* 2014;12:109.
17. Vellas B, Villars H, Abellan G, et al. Overview of the MNA--Its history and challenges. *The journal of nutrition, health & aging.* 2006;10(6):456-463; discussion 463-455.
18. Rubenstein LZ, Harker JO, Salva A, Guigoz Y, Vellas B. Screening for undernutrition in geriatric practice: developing the short-form mini-nutritional assessment (MNA-SF). *J Gerontol A Biol Sci Med Sci.* 2001;56(6):M366-372.
19. Kaiser MJ, Bauer JM, Ramsch C, et al. Validation of the Mini Nutritional Assessment short-form (MNA-SF): a practical tool for identification of nutritional status. *The journal of nutrition, health & aging.* 2009;13(9):782-788.

© 2021 Greysen SR et al. *JAMA Network Open.*

eTable 2: Adverse Events Reported

Participants, %	Control (n=118)	Intervention (n=115)	Overall (n=233)
Adverse events - Expected			
Not related	0	0	0
Possibly related	0	0	0
Related*	2 (1.7%)	2 (1.7%)	4 (1.7%)
Adverse events - Unexpected			
Not related	0	0	0
Possibly related**	1 (0.8%)	0	1 (0.4%)
Related	0	0	0
Serious Adverse events - Expected			
Not related	0	0	0
Possibly related	0	0	0
Related	0	0	0
Serious Adverse events - Unexpected			
Not related	0	0	0
Possibly related	0	0	0
Related	0	0	0

* All 4 Expected/Related events were skin rash at the site of watch wristband
** 1 Unexpected/Possibly related event was a fall; participant did not provide additional details

© 2021 Greysen SR et al. *JAMA Network Open.*

eTable 3: Adjusted Differences in Daily Steps Using Collected Data (No Imputations)

Primary Outcome	Overall (N=219)		Higher Social Engagement Subgroup (n=70)		Age 50-65 years Subgroup (n=51)	
	**Control (n=112)**	**Intervention (n=107)**	**Control (n=38)**	**Intervention (n=32)**	**Control (n=26)**	**Intervention (n=25)**
**Baseline**<br>Steps per day, mean (SD)	3898 (2582)	3765 (2866)	3484 (2415)	3869 (2967)	3430 (2605)	2540 (1397)
**Intervention period**<br>Steps per day, mean (SD)	3934 (2842)	3977 (2941)	3240 (2344)	4428 (2706)	3142 (2898)	2809 (1762)
**Main adjusted model**						
Difference relative to control and adjusted for baseline (95% CI)	-	206 (-321, 733)	-	1026 (345, 1707)	-	552 (-233, 1337)
P value	-	0.443	-	0.003	-	0.168

Abbreviations: SD, standard deviation; CI, confidence interval

*Main adjusted model adjusts for baseline step count, repeated measures, and has fixed effects for calendar month and study arm

© 2021 Greysen SR et al. *JAMA Network Open.*

eTable 4: Adjusted Differences in Daily Steps Using Collected Data (No Imputations) and Excluding Step Values < 1000

Primary Outcome	Overall (N=214)		Higher Social Engagement Subgroup (n=68)		Age 50-64 years Subgroup (n=50)	
	**Control (n=110)**	**Intervention (n=104)**	**Control (n=37)**	**Intervention (n=31)**	**Control (n=26)**	**Intervention (n=25)**
**Baseline**<br>Steps per day, mean (SD)	3934 (2591)	3815 (2889)	3540 (2422)	3949 (2981)	3430 (2605)	2588 (1406)
**Intervention period**<br>Steps per day, mean (SD)	4461 (2823)	4526 (2870)	3780 (2344)	4924 (2624)	3614 (2780)	3270 (1574)
**Main adjusted model**						
Difference relative to control and adjusted for baseline (95% CI)	-	141 (-347, 630)	-	815 (184, 1447)	-	513 (-180, 1206)
P value	-	0.570	-	0.011	-	0.147

Abbreviations: SD, standard deviation; CI, confidence interval

*Main adjusted model adjusts for baseline step count, repeated measures, and has fixed effects for calendar month and study arm

© 2021 Greysen SR et al. *JAMA Network Open.*
