# Supplementary material for: Effect of Behaviorally Designed Gamification With a Social Support Partner to Increase Mobility After Hospital Discharge: A Randomized Clinical Trial
Source: JAMA Netw Open. 2021 Mar 24;4(3):e210952. doi: 10.1001/jamanetworkopen.2021.0952 (PMC7991973; doi:10.1001/jamanetworkopen.2021.0952)
Supplement: Supplement 3. — Data Sharing Statement [file jamanetwopen-e210952-s003.pdf]

# Data Sharing Statement

Greysen. Effect of Behaviorally Designed Gamification With a Social Support Partner to Increase Mobility After Hospital Discharge. *JAMA Netw Open*. Published March 24, 2021. doi:10.1001/jamanetworkopen.2021.0952

**Data**
**Data available:** Yes
**Data types:** Deidentified participant data, Data dictionary
**How to access data:** ryan.greysen@pennmedicine.upenn.edu
**When available:** With publication

**Supporting Documents**
**Document types:** None

**Additional Information**
**Who can access the data:** Researchers whose proposed use of the data has been approved
**Types of analyses:** specified purpose
**Mechanisms of data availability:** After approval of a proposal, or with a signed data access agreement
